# Vasovagal reaction secondary to bladder overdistension in a dog undergoing a unique timeline of medical and surgical treatment for *Corynebacterium urealyticum* encrusting cystitis: a case report

**DOI:** 10.1186/s12917-021-03028-z

**Published:** 2021-09-28

**Authors:** Ryan F. Peiffer, Carly Iulo, Tessa LeCuyer, Timothy Bolton

**Affiliations:** 1 BluePearl Specialty and Emergency Pet Hospital, Sandy Springs, GA USA; 2 Upstate Vet Emergency and Specialty Care, Greenville, SC USA; 3 Department of Biomedical Science and Pathobiology, Virginia-Maryland Regional College of Veterinary Medicine, Virginia Tech University, Blacksburg, VA USA; 4 Department of Small Animal Clinical Sciences, Virginia-Maryland Regional College of Veterinary Medicine, Virginia Tech University, Blacksburg, VA USA

**Keywords:** Encrusting cystitis, *Corynebacterium urealyticum*, Vasovagal reaction, Surgery, Dog

## Abstract

**Background:**

*Corynebacterium urealyticum* urinary tract infections can result in a rarely reported condition called encrusting cystitis whereby plaque lesions form on and within the urinary bladder mucosa. Chronic lower urinary tract signs manifest subsequent to the infection-induced cystitis and plaque-induced decreased bladder wall distensibility. Because of the organism’s multidrug resistance and plaque forming capability, infection eradication can be difficult. While systemic antimicrobial therapy is the mainstay of treatment, adjunctive surgical debridement of plaques has been used with relative paucity in such cases, thereby limiting our understanding of this modality’s indications and success rate. Consequently, this report describes the successful eradication of *Corynebacterium urealyticum* encrusting cystitis utilizing a unique timeline of medical and surgical treatments. Additionally, this represents the first reported veterinary case of a vasovagal reaction due to bladder overdistension.

**Case presentation:**

A 6-year-old female spayed Miniature Schnauzer was evaluated for lower urinary tract clinical signs and diagnosed with *Corynebacterium urealyticum* encrusting cystitis. The infection was persistent despite prolonged courses of numerous oral antimicrobials and urinary acidification. A unique treatment timeline of intravenous vancomycin, intravesical gentamicin, and mid-course surgical debridement ultimately resulted in infection resolution. During surgery, while the urinary bladder was copiously flushed and distended with saline, the dog experienced an acute vasovagal reaction from which it fully recovered.

**Conclusions:**

Surgical debridement of bladder wall plaques should be considered a viable adjunctive therapy for *Corynebacterium urealyticum* encrusting cystitis cases failing to respond to systemic antibiotic therapy. The timing in which surgery was employed in this case, relative to concurrent treatment modalities, may be applicable in future cases of this disease as dictated on a case-by-case basis. If surgery is ultimately pursued, overdistension of the urinary bladder should be avoided, or at least minimized as much as possible, so as to prevent the possibility of a vasovagal reaction.

## Background

Encrusting cystitis is a chronic inflammatory condition of the urinary bladder characterized by a urease-producing bacterial infection, alkaline urine pH, and deposition of magnesium ammonium phosphate (struvite) precipitates on and within the urinary bladder mucosa [1]. These precipitates, coalescing to form encrustations or plaques, are extremely painful and produce a bladder wall with decreased distensibility [1]. In dogs and cats, encrusting cystitis is most commonly associated with the gram-positive bacillus *Corynebacterium urealyticum*; however, it has also been reported with other urease-producing bacteria such as *Staphylococcus pseudintermedius* [2–11].

Because of multi-drug resistance and the propensity to sequester within plaques, eradication of *Corynebacterium urealyticum* can be challenging [1, 12]. Treatment modalities employed in human and veterinary patients include systemic and intravesical antibiotic therapy, urinary acidification, and surgical debridement of bladder mucosa to remove calcified plaques [1–12]. Recent veterinary retrospective studies and case reports describe patients with *Corynebacterium urealyticum* urinary tract infections with and without encrusting cystitis [2–7]. The success rate for infection eradication amongst these 6 studies is 75% (24/32), with 75% (18/24) of the successfully treated cases the result of medical treatment only and 25% (6/24) the result of adjunctive surgical plaque debridement [2–7]. Of the 15 reported cases of encrusting cystitis, surgical debridement was only employed in 6 of them [2–7]. Thus, surgery is used infrequently in cases of *Corynebacterium urealyticum* encrusting cystitis, limiting our understanding of this modality’s indications and true success rate.

The goals of reporting this case are to: 1) describe the successful treatment of *Corynebacterium urealyticum* encrusting cystitis utilizing surgery as an adjunctive therapy; 2) detail a unique timeline of medical and surgical treatments; and 3) report the first incidence of a vasovagal reaction due to urinary bladder overdistension in a dog.

## Case presentation

A 6-year-old female spayed Miniature Schnauzer was referred for evaluation of lower urinary tract signs secondary to a persistent urinary tract infection. An initial urinalysis performed by the referring veterinarian revealed a pH of 8.0, > 50 white blood cells (WBC)/high powered field (hpf), 2 + magnesium ammonium phosphate (struvite) crystalluria, and rod bacteriuria. Failure to improve following a 10-day course of empiric cephalexin^1^ led to the identification of 4–5 urocystoliths on abdominal radiographs. A cystotomy with stone analysis diagnosed struvite urolithiasis, after which a stone prevention diet (Royal Canin® Urinary SO) was initiated. Despite these therapies, there was no improvement in clinical signs and the aforementioned urinalysis abnormalities persisted. A 10-day course of empiric enrofloxacin^2^ was subsequently initiated; however, due to the persistence of clinical signs, a urine culture was performed that lead to the identification of *Corynebacterium urealyticum*. Referral for treatment of the multidrug resistant urinary tract infection was pursued.

**Fig. 1:**
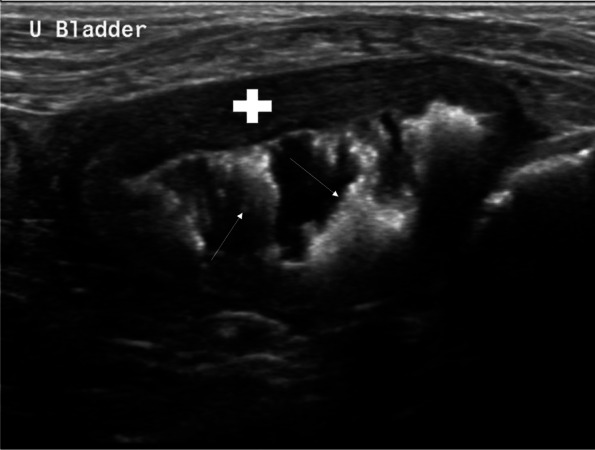
Ultrasonographic image of the urinary bladder, demonstrating severe, diffuse thickening of the wall (white plus sign) and adherent, mineralized plaques extending from the mucosa (white arrows)

At the time of referral, no physical examination abnormalities were detected; however, when walked outside, the dog postured frequently producing a small volume of bloody urine each time. Urinalysis revealed a pH of 8.5, 40–61 WBC/hpf, rare struvite crystals, and large numbers of rod bacteria. Ultrasonography of the urinary bladder demonstrated multifocal hyperechoic foci adhered to a diffusely thickened wall, consistent with encrusting cystitis [12] (Fig. 1).

Urine culture grew > 100,000 colony forming units (CFU)/mL of *Corynebacterium urealyticum*. *C. urealyticum* was identified by matrix-assisted laser desorption ionization-time of flight (MALDI-TOF) mass spectrometry^3^ and susceptibility testing was performed by determining the minimum inhibitory concentrations (MICs) of antimicrobial drugs by broth microdilution in Mueller–Hinton broth with lysed horse blood using a commercial plate.^4^ Following a 24-h incubation at 35ºC in ambient air, the MICs were read and interpreted on a commercial reader^5^ using Clinical Laboratory Standards Institute (CLSI) breakpoints programmed into the instrument’s software.^6^ Programmed breakpoints were based on breakpoints published in CLSI documents VET06 and VET08 [13, 14]. The organism was susceptible to rifampin, gentamicin, tetracyclines, and vancomycin (Culture #1 in Table 1). Despite low levels of urinary excretion, treatment with doxycycline^7^ 5 mg/kg PO q12h for 4 weeks was initiated based on in vitro susceptibility and in vivo efficacy [2]. Methionine^8^ 53 mg/kg PO q12h was additionally initiated for urinary acidification and the previously prescribed stone prevention diet was continued.

**Table 1:** *Corynebacterium Urealyticum* isolate breakpoints and MICs

	**Culture #1**		**Culture #2**		**Culture #3**		**Culture #4**
**Time From Referral**	Day 0		Day 30		Day 50		Day 76
**Specimen**	Urine (cystocentesis)		Urine (cystocentesis)		Urine (cystocentesis)		Bladder mucosa
**Result**	> 100 k CFU/mL	*C. urealyticum*	> 100 k CFU/mL	*C. urealyticum*	> 100 k CFU/mL	*C.urealyticum*	No growth
	MIC (μg/mL)	Reported Interpretation	MIC (μg/mL)	Reported Interpretation	MIC (μg/mL)	Reported Interpretation	
Amikacin	≤ 16	NI	≤ 16	NI	≤ 16	NI	
Amoxicillin-Clavulanate	> 8	*R*	> 8	*R*	> 8	*R*	
Ampicillin	> 256	NI	> 8	NI	> 8	NI	
Cefazolin	> 4	*R*	> 4	*R*	> 4	*R*	
Cefovecin	> 8	NI	> 8	NI	> 8	NI	
Cefpodoxime	> 8	NI	> 8	NI	> 8	NI	
Cephalothin	> 4	NI	> 4	NI	> 4	NI	
Chloramphenicol	≤ 8	NI	≤ 8	NI	≤ 8	NI	
Clindamycin	> 4	**R**	< = 0.5	**S**	> 4	**R**	
Doxycycline	0.5	**S**	> 0.5	NI	> 0.5	NI	
Enrofloxacin	> 4	NI	> 4	NI	> 4	NI	
Erythromycin	> 4	**R**	≤ 0.25	**S**	> 4	**R**	
Gentamicin	≤ 4	**S**	≤ 4	**S**	≤ 4	**S**	
Marbofloxacin	> 4	NI	> 4	NI	> 4	NI	
Minocycline	≤ 0.5	NI	> 2	NI	1	NI	
Nitrofurantoin	> 64	NI	> 64	NI	> 64	NI	
Oxacillin	> 2	NI	> 2	NI	> 2	NI	
Penicillin	> 8	**R**	> 8	**R**	> 8	**R**	
Pradofloxacin	> 2	NI	> 2	NI	> 2	NI	
Rifampin	≤ 1	**S**	≤ 1	**S**	≤ 1	**S**	
Tetracycline	≤ 2	**S**	> 1	NI	> 1	NI	
Trimethoprim Sulfa	> 8	**R**	> 4	**R**	> 8	NI	
Vancomycin	≤ 1	**S**	≤ 1	**S**	≤ 1	**S**	

*Italics* = VET08 Staphylococcus breakpoints

Bold = VET06 Coryneform breakpoints

*S* Susceptible, *I* Intermediate, *R* Resistant, *NI* No interpretation available

At a recheck 4 weeks later, the lower urinary tract signs were improved (less frequent stranguria, pollakiuria, hematuria) but unresolved. Alkalinuria, pyuria, and bacteriuria persisted on urinalysis and *Corynebacterium urealyticum* was cultured from the urine (Culture #2 in Table 1). Treatment with chloramphenicol^9^ 27 mg/kg PO q8h for 4 weeks was initiated based on previously documented *Corynebacterium urealyticum* in vitro susceptibility to this antibiotic [3]. Although this treatment initially resulted in some abatement of clinical signs, the lower urinary tract symptoms ultimately worsened on treatment. *C. urealyticum* was again cultured from the urine (Culture #3 in Table 1).

The failure of outpatient treatment to clear the infection and owner decision to pursue euthanasia if unresolved prompted hospitalization. Vancomycin^10^ 15 mg/kg IV q8h and gentamicin^11^ 6 mg/kg intravesical as a single dose were administered [5]. An indwelling Foley urinary catheter was placed to facilitate gentamicin infusion into the bladder, with instillation immediately causing appreciable discomfort. Following infusion, the catheter was occluded to prevent the dog from voiding. The intended dwell time was 6 h; however, urine leaked around the catheter, thus the volume of gentamicin that remained within the bladder during the dwell time was unknown. A second dose of intravesical gentamicin originally intended for administration 24 h following the initial dose was aborted because the urinary catheter became obstructed with debris and was subsequently removed. Despite 6 days of intravenous vancomycin and a single dose of intravesical gentamicin, the lower urinary tract signs persisted. Therefore, surgical debridement of the urinary bladder was pursued. A urinalysis performed on the day of surgery revealed a pH of 7.0, 8–15 white blood cells/hpf, and no struvite crystalluria or bacteriuria, all improvements compared to prior urinalyses. The markedly improved pyuria and lack of bacteria indicated in vivo vancomycin efficacy.

A caudal midline celiotomy and ventral cystotomy were performed. The urinary bladder was hyperemic and thickened on palpation. Following exposure of the urinary bladder lumen, multiple grey-green calcified plaques were visible on the mucosal surface, with most concentrated along the ventral surface and around each ureteral papilla. A full thickness bladder wall biopsy was submitted for histologic analysis and a sample of mucosa with calcified plaques was submitted for bacterial culture. The mucosal plaques were debrided using a #15 blade. Copious flushing and subsequent maximal distension of the urinary bladder with a 10-Fr red rubber catheter and sterile saline was then performed, during which the dog experienced acute bradycardia and hypotension progressing to asystole. The celiotomy incision was extended cranially to the xyphoid, whereby a diaphragmatic incision allowing access to the thoracic cavity was performed. Cardiac massage and resuscitative drug therapy (atropine) was administered, resulting rapidly in patient revival. Once stabilized, the diaphragm, urinary bladder, and abdominal incisions were closed. Prior to anesthetic recovery, an indwelling urinary catheter was placed to monitor urine appearance and output post-operatively. Anesthetic recovery was uneventful, with all normal vital parameters.

**Fig. 2:**
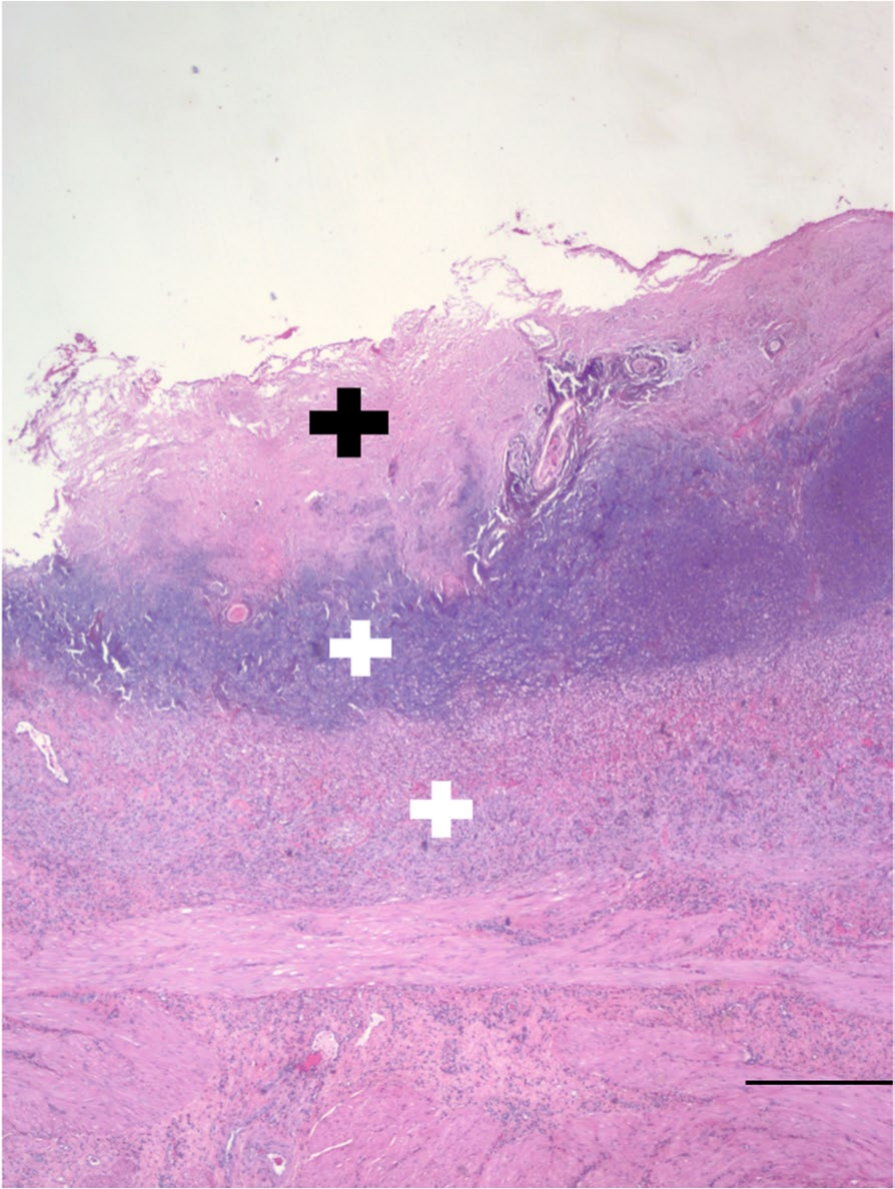
Photomicrograph of a section of urinary bladder wall demonstrating mineralized crusts on the luminal (mucosal) surface (black plus sign) and suppurative inflammation within the mucosa, submucosa, and muscularis layers (white plus signs). Hematoxylin and eosin stain; bar = 500 µm. Acquired image resolution = 96 dpi. Processed image resolution = 150 dpi

Histologic examination of the urinary bladder wall revealed severe suppurative and necrotizing cystitis of the mucosal and submucosal layers. Mineralization of these same layers was also present (Fig. 2,^12^,^13^). These histopathologic findings are identical to those described in previous reports of encrusting cystitis [3, 5]. Culture of the bladder mucosa containing calcified plaques was negative for bacterial growth (Culture #4 in Table 1), indicating adequate penetration of vancomycin into the diseased urinary bladder wall.

Postsurgical care included the administration of intravenous fluids, vancomycin 15 mg/kg IV q8h, methionine 53 mg/kg PO q12h, and meloxicam^14^ 0.1 mg/kg PO q24h. Urine draining from the indwelling urinary catheter immediately following surgery was serosanguinous; however, it was grossly normal the following morning and the catheter was removed. The other lower urinary tract signs (stranguria and pollakiuria) were dramatically improved at the time of discharge 8 days after surgery. The dog received a total of 14 days of vancomycin therapy (6 days prior to and 8 days after surgery) and one dose of intravesical gentamicin during hospitalization. A urine culture performed 5 weeks following treatment was negative for bacterial growth and the dog was lower urinary tract symptom free. A phone update with the owner 3 and 9 months after treatment revealed the dog to still be clinically normal.

## Discussion and conclusions

In humans, *Corynebacterium urealyticum* is a significant nosocomial pathogen with established risk factors for infection and subsequent encrusting cystitis development. These include historical urologic disease (bladder trauma, local neoplasia, micturition disorders, or urinary tract infection), prior broad-spectrum antibiotic use, systemic immunosuppression, or a recent urologic procedure such as cystoscopy, surgery, or catheterization [15]. Similar risk factors have been described for *Corynebacterium urealyticum* urinary tract infections in the veterinary literature; however, in not all cases has such a factor been identified [2–11]. The patient described in this case report underwent surgery to remove bladder stones and received antibiotic treatment for a urinary tract infection prior to documentation of the *Corynebacterium urealyticum* infection. However, because no urine culture was performed prior to either of the aforementioned treatments, it is possible that an infection with *Corynebacterium urealyticum* was present at the initial evaluation by the referring veterinarian. If this were true, no known risk factor for *Corynebacterium urealyticum* infection development would be present in this case.

Prolonged (> 1 month) culture-based antibiotic therapy is a crucial treatment modality required for the resolution of *Corynebacterium urealyticum* infections in both human and veterinary patients. In several of the initial reports of this condition in humans, both tetracyclines and fluoroquinolones were utilized with success; however, because of immutable resistance to some antibiotic classes (β-lactams, cephalosporins, and aminoglycosides) and variable yet growing resistance to other classes (tetracyclines and fluoroquinolones), glycopeptides like vancomycin and teicoplanin are currently the first-line therapy in most human cases [16, 17]. Conversely, in veterinary patients, glycopeptides are rarely used and likewise not considered first-line therapy in this disease or others due to concern for the development of vancomycin resistant organisms [18]. Furthermore, urinary tract infections caused by *Corynebacterium urealyticum* have historically demonstrated in vivo efficacy and/or in vitro susceptibility to numerous other antibiotic classes such as tetracyclines, fluoroquinolones, and chloramphenicol [2, 3]. Selecting an appropriate antimicrobial for veterinary patients is additionally complicated by the lack of species-specific breakpoints for *Corynebacterium* spp. The CLSI VET06 contains breakpoints for coryneform bacteria, but these are extrapolations from human breakpoints which are not necessarily suitable for dogs and cats [14]. For these reasons and based on the urine cultures performed during referral care, the dog in this report was treated initially with doxycycline and subsequently chloramphenicol in an effort to eradicate the infection. Only after the persistence of clinical signs on both antibiotics, the lack of infection resolution on urine culture, and the limited antibiotic choices was vancomycin therapy decided upon.

In addition to proper antibiotic therapy, humans with a *Corynebacterium urealyticum* urinary tract infection resulting in encrusting cystitis often undergo surgical debridement of plaque lesions as part of the multi-modal treatment approach to this disease [19]. This is routinely recommended because plaques harbor large quantities of bacteria and prevent adequate antibiotic penetration, thus limiting in vivo antibiotic efficacy [12]. Because plaque debridement is not currently the standard of care in veterinary patients, it has only been utilized in 40% (6/15) of recently reported cases of encrusting cystitis in the dog and cat [2–7]. In these 6 cases, encrusting cystitis resolved in 83% when plaque debridement was a part of the treatment protocol [2–4]. In all successfully debrided cases, including the one detailed in this case report, patients were receiving antibiotics concurrently, precluding any inference that surgery was the sole reason for the positive outcomes [2–4]. However, this case report does add to the paucity of *Corynebacterium urealyticum* encrusting cystitis cases utilizing plaque debridement and having a successful outcome when this adjunctive modality is part of the treatment plan. Studies evaluating specific criteria to determine which cases may benefit from or require concurrent plaque debridement are needed; however, to date, it would seem that urinary tract obstruction (ureteral and/or urethral) or a failing response to medical therapy would fulfill the criteria [4]. At what juncture in a case medical therapy would be considered a failure also needs to be better defined, but would likely entail progression towards antibiotics traditionally reserved for multidrug-resistant infections, such as carbapenems and vancomycin [20].

Following the failure of multiple, outpatient, culture-based antibiotic treatments to resolve the infection, the dog in this report was hospitalized for more aggressive care. Based on the available data and most recent urine culture in vitro susceptibility report (Culture #3 in Table 1), intravenous vancomycin in combination with intravesical gentamicin was decided upon [3, 5]. Despite 6 days of vancomycin therapy and a single dose of intravesical gentamicin, the dog remained severely pollakiuric and hematuric. Thus, the decision was made to proceed with surgical debridement. The decision to proceed with surgery at that point as opposed to at the commencement of hospitalization was dictated by numerous interrelated factors based in both scientific evidence and clinical acumen. First, ~ 56% of recent encrusting cystitis cases resolved with medical therapy alone, thus the hope from hospitalization outset was that this more aggressive antibiotic treatment would be successful [2–7]. Second, despite the 6 days of hospitalized treatment, the dog continued to have severe lower urinary tract signs, raising concerns that continued medical treatment alone would be insufficient to resolve the infection. Third, as demonstrated in a recent report, there was concern that in vitro vancomycin susceptibility would not correlate with in vivo efficacy, potentially resulting in a vancomycin resistant organism that could not be subsequently cleared even with surgery [5]. Thus, the 6-day treatment window prior to surgery indirectly served as a ‘trial’ to determine if the urinary tract infection could be cleared, thereby demonstrating in vivo vancomycin efficacy and making surgery more likely to result in a successful outcome. A urinalysis performed on the day of surgery revealed a marked reduction in pyuria and resolution of bacteriuria, increasing the likelihood that surgical debridement of plaques would be efficacious at this point in the case. Lastly, the owner indicated a decision to euthanize the dog if infection resolution was not achieved. Because of conflicting clinical and laboratory evidence at the 6-day mark as to whether the infection was likely to resolve with continued medical therapy alone and the concern that without surgery the infection could remain sequestered within the bladder mucosal plaques, surgery was elected at the midway point of medical treatment [15]. Based on these four aforementioned factors, this case underwent a unique timeline of treatments, starting with 6 days of intravenous vancomycin and a single dose of intravesical gentamicin, followed by mid-course surgical plaque debridement and 8 more days of vancomycin following surgery. The end result was infection resolution, with the dog remaining symptom free at last recheck (9 months).

Intravesical antibiotic therapy was utilized in this case based on a recent report of its success in the treatment of *Corynebacterium urealyticum* encrusting cystitis in a dog [5]. This route of therapy allows for the direct delivery of drug to the infection site, resulting in high local concentrations and minimal systemic absorption. Gentamicin was the antibiotic instilled into the urinary bladder in this particular case based on its lack of substantial systemic absorption in both humans and dogs as well as its success as the intravesical antibiotic of choice in a previous veterinary case [5, 21, 22]. During gentamicin instillation, the dog experienced unexpected discomfort, potentially a result of the low drug pH (4.5) and/or overdistension of a urinary bladder with already decreased distensibility from encrusting cystitis. In addition to antimicrobial effects, the acidity of the gentamicin solution may have assisted with infection eradication. The leakage of urine around and occlusion of the urinary catheter with debris highlighted the difficulty of maintaining such a catheter in cases of encrusting cystitis, potentially limiting the utility of his treatment modality. For these reasons, the dwell time for the initial gentamicin dose was limited and a second dose was not administered, unlike in a previous veterinary case [5]. Notably, the successful use of intravesical gentamicin in this prior case was preceded by failed surgical debridement. While the theoretical benefits of this locally targeted therapy are apparent, its role in the resolution of the resistant infection in this case and in the future management of veterinary encrusting cystitis cases is less clear.

An acute decline in blood pressure, heart rate, and cardiac output triggered by activation of the vagus nerve is termed a vasovagal reaction. In humans, this can be elicited by various stimuli, such as anxiety, pain, and bladder overdistension [23–25]. In the dog of this report, acute bradycardia and hypotension progressing to sinus arrest during surgery unexpectedly occurred. There were no identified comorbidities and the dog, until the point of acute decompensation, was cardiovascularly stable under anesthesia. Interestingly, at the time of decompensation, the urinary bladder was being copiously flushed and maximally distended with saline. Consequently, parasympathetic stimulation initiated by bladder overdistension causing a vasovagal reaction was strongly suspected to be the inciting cause for the acute anesthetic decompensation. The rapid response to parasympatholytic drug therapy (atropine) provides strong support for a vasovagal reaction as the culprit. In cases of *Corynebacterium urealyticum* encrusting cystitis undergoing a cystotomy for surgical debridement of plaques, careful consideration should be given to the degree of bladder distension in order to avoid this life-threatening reaction from possibly occurring. This represents the first known report of a vasovagal reaction due to bladder overdistension in the dog.

Several limitations to the management of this case must be acknowledged. First, it is unknown which treatment modality was the primary driver for infection eradication. It is possible that with continued (> 6 days) vancomycin therapy alone, the infection would have been cleared and surgery would not have been needed. Second, an alternative cause for the cardiac arrest cannot be excluded. Although no comorbidities were apparent on physical examination or found during pre-anesthetic diagnostic workup, an occult disease could not be excluded. Despite this possibility, the vasovagal reaction was suspected to be surgery and not anesthesia induced for two reasons: 1) the dog was at stable plane of anesthesia for over 2.5 h prior to decompensation and 2) it occurred at the time copious bladder flushing and distension was occurring. Lastly, the unique timeline of treatment in this dog was dictated by many factors unique to this case. Thus, this timeline of treatment should not be attempted nor considered prudent in every case of encrusting cystitis.

*Corynebacterium urealyticum* encrusting cystitis is a chronic disease, often requiring prolonged antibiotic treatment and less frequent surgical plaque debridement for infection resolution. Both clinical signs and results of urine culture and sensitivity should be monitored vigilantly to detect the development of progressive antibiotic resistance of this already multidrug resistant organism. As antibiotic options decline and clinical disease persists, surgical bladder debridement should be considered an adjunctive therapy that may assist in resolving the infection. Although an uncommon disease entity, both veterinary specialists and general practitioners are going to be challenged by the difficulties of treating this multidrug resistant infection. This case report not only adds to the small number *Corynebacterium urealyticum* encrusting cystitis cases cured utilizing surgery as an adjunctive treatment but also is the first reported case of a vasovagal reaction secondary to bladder overdistension in the dog.

## Data Availability

All data generated or analyzed during this study are included in this published article.

## Abbreviations

WBC: White blood cells
hpf: High-powered field
CFU: Colony forming unit
MIC: Minimum inhibitory concentration
CLSI: Clinical Laboratory Standards Institute
PO: Per os
IV: Intravenous

## Footnotes

1 Keflex®, Ascend Laboratories, Parsippany, New Jersey

2 Baytril®, Bayer Healthcare, Shawnee Mission, Kansas

3 Bruker MALDI Biotyper, Bruker Daltonics Inc., Billerca, Massachusetts

4 Sensititre™ Companion Gram Positive COMPGP1F Vet AST Plate, ThermoFisher Scientific, Waltham, Massachusetts

5 Sensititre™ Vizion™ Digital MIC Viewer, ThermoFisher Scientific, Waltham, Massachusetts

6 Sensititre™ SWIN™ Software, ThermoFisher Scientific, Waltham, Massachusetts

7 Vibramycin®, West-Ward Pharmaceutical, Eatontown, New Jersey

8 DL-Methionine®, V.E.T. Pharmaceutical, Miramar, Florida

9 Viceton®, Bimeda, Le Sueur, Minnesota

10 Vancocin®, Alvogen, Pine Brook, New Jersey

11 Garamycin®, MWI, Boise, Idaho

12 EOS Rebel T2i (via Olympus BX41 microscope), Canon U.S.A. Inc., Melville, New York

13 EOS Utility 2, version 2.14.20.0, Canon U.S.A Inc., Melville, New York

14 Metacam®, Boehringer Ingelheim, St. Joseph, Missouri
